# AGING IS VERY PERSONAL: INTERPROFESSIONAL GERONTOLOGY COURSE IMPROVING STUDENT ATTITUDES TOWARD OLDER ADULTS

**DOI:** 10.1093/geroni/igz038.542

**Published:** 2019-11-08

**Authors:** Jason T Garbarino

**Affiliations:** University of Vermont, Burlington, Vermont, United States

## Abstract

Educational programs that foster the development of a robust healthcare workforce committed to the provision of exemplary care of older adults is vital. The Aging is Very Personal (AIVP) service learning gerontology course has demonstrated the ability to foster future student interest and improved attitudes towards working with older adults. The AIVP program provides mutual benefit for undergraduate students from a variety of health science majors and older adult resident volunteers at local senior living facilities. For students, AIVP serves as direct insight into the lived experience of aging among community older adults. Students are provided the opportunity to practice communication skills, relationship-building skills, and gain an understanding of the multitude of diverse needs within this population. Older adults who volunteer to participate in the activity are provided with the opportunity to speak to and actively engage with students and feel empowered by the opportunity to provide valuable life guidance. This presentation will provide a curricular overview of the steps required to construct, implement, and evaluate an interprofessional gerontology course. A review of student learning objectives, service-learning program construction, selected course topics, and student assignments will be presented. Attitudes and future interest in working with older adults measured in the initial interprofessional student cohort (n=106) will be presented. A pre-established, validated tool utilized to effectively measure student attitudes and interest pre/post-course participation will be reviewed.

